# Endorsement of Criminal Behavior Amongst Offenders: Implications for DSM-5 Gambling Disorder

**DOI:** 10.1007/s10899-015-9540-3

**Published:** 2015-03-28

**Authors:** Nigel E. Turner, Randy Stinchfield, John McCready, Steven McAvoy, Peter Ferentzy

**Affiliations:** 1 Centre for Addiction and Mental Health, 33 Russell Street, Rm. T524, Toronto, ON M5S 1R8 Canada; 2 Dalla Lana School of Public Health, University of Toronto, Toronto, ON Canada; 3 Department of Psychiatry, University of Minnesota Medical School, Minneapolis, MN USA; 4 Healthy Horizons Consulting, Toronto, ON Canada

**Keywords:** Problem gambling, Offenders, DSM-5, Illegal acts, Convergent validity

## Abstract

The fifth edition of the diagnostic and statistical manual (DSM) has changed the scoring threshold for a gambling disorder (GD) from five criteria to four and eliminated the illegal acts criterion. The impact of these changes was examined with data from a correctional population (N = 676) in Ontario, Canada. The offenders completed a self-report survey that included the Canadian problem gambling index, the South Oaks Gambling Screen and the DSM-IV criteria. Changing the threshold from 5 to 4 improved the convergent validity for GD and resulted in an increase in the percentage of offenders diagnosed with a GD from 7.4 to 10.2 %. The results also indicate that the illegal acts criterion contributes to the convergent validity of GD. The evidence supports the change in the threshold from five to four, but also reinforces the importance of examining illegal acts when dealing with an offender population. The incorporation of illegal acts into the “lying to others” criteria appears to make up, to some extent, for the removal of the illegal acts criterion.

## Introduction

In 2013, the American Psychiatric Association published the 5th edition of diagnostic and statistical manual (DSM-5) (American Psychiatric Association 2013; Grant 2013). The DSM-5 included several changes to the criteria used to diagnose pathological gambling (American Psychiatric Association 2013; Grant 2013). The disorder was renamed gambling disorder (GD) instead of pathological gambling. It was reclassified into a new category called Substance-related and Addictive Disorders. The diagnosis of GD also now includes a range in severity from mild to severe (Grant 2013). More important for the current paper, the cut score for a diagnosis was changed from the endorsement of five or more of the diagnostic criteria to the endorsement of four or more. In addition, one of the ten criteria, committing “illegal acts such as forgery, fraud, theft, or embezzlement to finance gambling” was eliminated from the DSM-5 scale as a free-standing criterion and is now subsumed under the “lying to others” criterion. The current paper examines the impact of the change in the cut point of GD and elimination of the illegal acts criterion with an offender sample in Ontario.

The fourth edition of the diagnostic and statistical manual (DSM-IV-TR) consisted of a list of ten criteria that describe common symptoms of disordered gambling (American Psychiatric Association 2000). A number of studies have found that the cut point of five was too conservative and may have underestimated the number of people who suffer from the disorder (Cox et al. 2004; Ferentzy and Turner 2013; Jimenez-Murcia et al. 2009; Lesieur and Rosenthal 1991; Stinchfield 2003; Stinchfield et al. 2005; Turner et al. 2009, 2013; Zimmerman et al. 2006). For example, Cox et al. (2004) found that the prevalence rate (2.6 %) based on the South Oaks Gambling Screen (SOGS; Lesieur and Blume 1987) was twice as high as the estimated prevalence rate based on the DSM-IV (1.3 %). Furthermore, they argue that “subthreshold individuals significantly differed from recreational gamblers and more closely approximated the characteristics displayed by pathological gamblers” (p. 258). Similarly, in a study of offenders using various measures, Turner et al. (2009) found that 6.3 % of offenders were identified as pathological gamblers using the DSM-IV criteria whereas the SOGS (Lesieur and Blume 1987) indicated that 13.0 % were probable pathological gamblers, and the Problem Gambling Severity Index (CPGI/PGSI; (Ferris and Wynne 2001) indicated that 9.4 % had a severe problem gambling. Caution must be exercised in the interpretation of these numbers, however. It could be argued that the SOGS or the CPGI are overly inclusive and yield too many false positives. Conversely, other studies report that the DSM-IV cut off score of five of ten criteria often results in false negatives, and thus some investigators have suggested changing the cut score from five to four (Jimenez-Murcia et al. 2009; Lesieur and Rosenthal 1991; Stinchfield 2003; Stinchfield et al. 2005).

Other researchers have examined the individual criterion and have concluded that the “illegal acts” criterion could be eliminated as a separate criterion because it adds little to diagnostic accuracy (Petry et al. 2013; Strong and Kahler 2007; Zimmerman et al. 2006) and does not contribute to the distinction of case versus non-case (Zimmerman et al. 2006). In addition, a number of large epidemiological surveys of gambling behavior in the United States and United Kingdom (e.g., McBride et al. 2010; Orford et al. 2003; Strong and Kahler 2007; Toce-Gerstein et al. 2003) have found that the illegal acts criterion was rarely endorsed unless the participant had also endorsed a number of other criteria (Petry et al. 2013) and hence that its elimination would not substantively affect the prevalence rate of disordered gambling. However, these analyses also suggested that the illegal acts criterion is most often present in individuals with the most severe form of the disorder (Strong and Kahler 2007) and therefore might be important in determining severity.

One limitation with the previous studies is that they have been based on data from clinical and general population studies of gamblers. It is likely that at least some of the people with gambling disorders who have committed criminal acts to support their gambling would be found in prison rather than treatment. A meta-analysis of research with correctional samples by Williams et al. (2005) found prevalence rates combining subclinical problem and pathological gambling ranging from 17 to 60 %, with an average of approximately 33 %. Turner et al. (2009) found that the illegal acts criterion was endorsed by 43 % of pathological gamblers (Turner et al. 2009), which is considerably more than the 25 % that was reported by Zimmerman et al. (2006) in a clinical population. It is possible that illegal acts may actually be more important when dealing with problem gamblers in correctional populations. To examine this issue we combined two data sets collected by Turner and colleagues (Turner et al. 2009, 2013). These data were collected from 2005 to 2012 from 676 incarcerated offenders in Ontario. The study used DSM-IV criteria as well as the CPGI/PGSI (Ferris and Wynne 2001), SOGS (Lesieur and Blume 1987), gambling frequency, and a measure of harmful consequences (see Turner et al. 2006). It was hypothesized that in a correctional population, the illegal acts criterion would be much more important in identifying pathological gamblers.

Given the recent changes to DSM-5, it is important to determine whether these revisions will improve diagnostic accuracy over DSM-IV diagnostic criteria for a gambling disorder. The following three questions were examined: (1) How is the rate of gambling disorders in a correctional population affected by the change in the criteria? (2) How do these changes in the DSM-5 affect convergent validity? and (3) Does the illegal acts criterion affect the classification of the client as having a mild, medium or severe gambling disorder?

## Methods

### Participants

The participants were drawn from two studies of offenders in Ontario. The first sample (Turner et al. 2009) consisted of 254 male federal offenders in an assessment unit of the Correctional Service of Canada (completion rate 39 %). The second sample (Turner et al. 2013) consisted of 422 offenders drawn from three provincial institutions and seven federal institutions (completion rate of 61.5 %). The 11 institutions selected provided us with a comprehensive overview of the types of correctional facilities in Southern Ontario. Federal institutions included all three security levels (minimum, medium, maximum) of the Correctional Service of Canada (CSC). In Canada, a federal offence is defined as a custodial sentence of 2 years or more. Offenders sentenced to <2 years are housed in separate provincial facilities.

The two data sets were merged into a single data-base. The combined sample was 676 (*M* = 635; *F* = 41), and included 555 federal offenders and 121 provincial offenders. The median sentence length was 156 weeks. The offenders ranged from 18 to 82 years of age with an average age of 37.1 years (*SD* = 12.0). Provincial offenders (M = 34.0, *SD* = 11.0) were significantly younger than federal offenders (M = 37.7, *SD* = 12.2), *t* (665) = 3.1, *p* < 0.01.

### Measures

The questionnaire package for these two studies was essentially the same and was derived from a questionnaire package used by Turner et al. (2006, 2008) to study severe problem gambling in the general population. Severe problem gambling was assessed using the SOGS (Lesieur and Blume 1987) framed in terms of past year prior to incarceration (*a* = 0.87), a questionnaire based on the 4th edition of the diagnostic and statistical manual (DSM-IV-TR; *a* = 0.85) developed by us that we have used in several previous studies (Turner et al. 2006, 2008), and the CPGI/PGSI [(Ferris and Wynne 2001); *a* = 0.93]. A SOGS score of five or more is interpreted as a probable pathological gambler and a CPGI/PGSI score of eight or more is classed as a severe problem gambler. The internal consistency statistics reported are those observed in our previous studies (Turner et al. 2006, 2008). In addition, we used a 12-item harmful consequences scale (Turner et al. 2006, 2008) which asked participants to rate, on a seven-point scale, harmful consequences experienced as a result of their gambling (e.g., “Has gambling caused you any problems with your physical health?” “Has gambling caused you any problems with your social relationships?”; *a* = 0.96). As reported in Turner et al. (2006, 2008), harmful consequences had strong correlations with both the DSM-IV, rho = 0.78, and the SOGS, rho = 0.72. In addition, participants were asked how often they played each of 16 different types of games. These measures were all framed in terms of their gambling prior to incarceration (see (Lesieur and Blume 1987) for details). A correctional file review was carried out to verify demographic data and criminal history information. The harmful consequences scale and CPGI/PGSI have no questions that specifically ask about illegal acts. The SOGS has a question that indirectly addresses one illegal behavior, (“passing bad checks”), but no direct statement about illegal acts.

### Procedures

The project was reviewed by the research ethics boards of the Centre for Addiction and Mental Health and Correctional Service of Canada. All participants read and signed a consent form indicating that their participation in the study was voluntary, and that they could withdraw at any time without any negative repercussions. The participants completed the questionnaires in small groups of between 2 and 10 people, at separate desks spaced well apart. Participants completed a series of questionnaires designed to screen for severe problem gambling and to examine various aspects of their gambling behavior. The researchers assisted anyone requesting clarification on the consent form or questionnaires. For sample 1, participants were recruited during a mandatory institutional orientation session (Turner et al. 2009) so that all offenders admitted to the institution during the study period were invited to participate. For sample 2, the participants were randomly selected from the institutional population list, invited to attend an information session, and then asked about volunteering for the study (Turner et al. 2013).

## Results

The first analysis examined the endorsement rate of the various DSM-IV criteria. Table 1 shows the overall endorsement rate and also breaks it down by DSM-IV diagnostic category. As shown, criterion 8 (illegal acts) is one of the least often endorsed, even amongst offenders. Criteria 9 (jeopardizing a relationship, job, education, or career) and 10 (relying on others for money), however, were endorsed less often in total than illegal acts. The overall alpha for the criteria was 0.86; all criteria had substantial item total correlations ranging from 0.49 to 0.60. The illegal acts criterion did have the lowest item total correlation: 0.49. However, the alpha if deleted was lower for all ten criteria indicating that all of the criteria contributed to reliability of the scale.

**Table 1:** Corrected item totals and endorsement rates for each of the ten criteria on the DSM-IV

	Corrected item-total correlation	Alpha if deleted	1–4 criteria endorsed	5+ criteria endorsed	Total criteria endorsed
			*N* = 161	*N* = 51	*N* = 676
1. Are you preoccupied with gambling?	0.60	0.84	26.1 %	76.0 %	11.8 %
2. Do you find that you need to gamble with increasing amounts of money in order to achieve the level of excitement you want?	0.54	0.85	24.8 %	60.0 %	10.4 %
3. Over the past 12 months, if you have made efforts to control your gambling, or to cut back on it, or to stop gambling altogether, have you ever found that you have been repeatedly unsuccessful?	0.51	0.85	26.7 %	68.0 %	11.4 %
4. Do you find that you are restless or irritable when you attempt to cut down or stop gambling?	0.58	0.84	7.5 %	56.0 %	5.9 %
5. Do you find that you gamble as a way to escape from your problems, or to relieve feelings of helplessness, or guilt, or anxiety or depression?	0.56	0.84	24.8 %	72.0 %	11.2 %
6. After you have lost money gambling, do you often return another day to get even? (Do you chase your losses?)	0.60	0.84	49.7 %	88.0 %	18.3 %
7. Do you lie to your family members, or therapist, or others in order to conceal the extent of your involvement with gambling?	0.69	0.83	9.9 %	82.0 %	8.4 %
8. Over the past 12 months, have you ever committed illegal acts such as forgery, fraud, theft or other embezzlement in order to finance your gambling?	0.49	0.85	9.9 %	44.0 %	5.6 %
9. Over the past 12 months, have you jeopardised or lost a significant relationship, job, educational or career opportunity because of your gambling?	0.58	0.84	5.0 %	54.0 %	5.2 %
10. Do you find that you rely on others to provide you with money when you are in a desperate financial situation caused by gambling?	0.57	0.84	4.3 %	56.0 %	5.2 %

The overall Cronbach’s alpha for the entire scale was 0.86

The effect of the two rule changes on the percentage of offenders with a gambling disorder is shown in Table 2. The percentage of offenders identified as having a gambling disorder increased from 7.4 to 10.2 % using a cut score of four rather than five. Removing illegal acts, however, resulted in a reduction of the percentage from 7.4 to 6.7 % if the cut off was five or more, and from 10.2 to 9.3 % if the cut off was four or more.

**Table 2:** Effect of rule changes on the percentage of disordered gambling in the offender population

Descriptive statistics	Number of disordered gamblers	Percent (*N* = 676)
5 of 10: with the illegal acts criterion	50	7.4
5 of 9: without the illegal acts criterion	45	6.7
4 of 10: with the illegal acts criterion	69	10.2
4 of 9: without the illegal acts criterion	63	9.3

Table 3 shows cross tabulations of the DSM-IV categorization with the various other possible DSM-5 models. Dropping illegal acts but retaining the cut off of five resulted in five offenders (10 %)—previously included as pathological gamblers—*not* being included in that same category. As expected, using a cut score of four increased the number of people who were classified as having a gambling disorder. As shown in Table 3, decreasing the cut score to four resulted in an increase from 50 to 63 (+26 %) in the number of offenders diagnosed with disordered gambling if illegal acts was removed, and an increase from 50 to 69 (+38 %) if illegal acts was retained. Table 4 shows the cross tabulation of the 4 of 9 model (excluding illegal acts) with the 4 of 10 model (with illegal acts). The removal of illegal acts produced a decrease in identified gambling disorders from 69 to 63.

**Table 3:** Cross tabulations of the current standard of 5 of 10 including the illegal acts criterion with the various other possible models with or without the illegal acts criterion (criterion 8)

		Current DSM-IV standard of 5 of 10 including the illegal acts criterion		
		NGD	GD	Total
5 of 9	NGD	626	5	631
without illegal acts	GD	0	45	45
4 of 10	NGD	607	0	607
with illegal acts	GD	19	50	69
4 of 9	NGD	613	0	613
without illegal acts	GD	13	50	63
Total		626	50	676

*GD* gambling disorder based on that criteria, *NGD* no gambling disorder

**Table 4:** Cross tabulation of DSM-5 cut point of 4, with and without the illegal acts criterion

	With the illegal acts criterion		
	NGD	GD	Total
*Without the illegal acts criterion*			
NGD	607	6	613
GD	0	63	63
*Total*			
	607	69	676

*GD* gambling disorder based on that criteria, *NGD* no gambling disorder

As noted above, using a cut off of four with illegal acts, six more people were identified as having a gambling disorder than without illegal acts. If these additional cases represent actual cases, then the inclusion of illegal acts for case versus non-case categorization should yield higher convergent validity correlations than without illegal acts. Table 5 shows the correlation of the gambling frequency, SOGS, CPGI/PGSI, and a harmful consequences measure with the original DSM criteria, with either a cut score of four or five, and with or without illegal acts. It also shows the correlations of the various cut scores with the same variables. In terms of correlations, a cut off of four with or without illegal acts produced higher correlations than a cut off of five. However, the DSM scoring with illegal acts included had somewhat higher correlations with the CPGI/PGSI total score, gambling frequency, SOGS scores, and harmful consequences than without illegal acts. In addition, the kappa for the CPGI/PGSI (eight or more), and the SOGS (five or more) was highest for the 4 of 10 model that included illegal acts. By including illegal acts the correlation between the CPGI/PGSI and the DSM-5 increased from 0.671 to 0.696 and this difference accounts for 3.4 % of the variance. The change from the DSM-IV (5 of 10 criterion) to the revised DSM-5 (4 of 9 criterion) was an increase from 0.654 to 0.671 and this difference accounts for 2.3 % of the variance. Similarly, the highest convergent validity coefficients for the SOGS and for harmful consequences were found for a DSM-5 total that included the illegal acts criterion (4 of 10 criterion).

**Table 5:** The effect of the illegal acts criterion and different cut points on convergent correlations with CPGI/PGSI, SOGS, harmful consequences, and gambling frequency

	5 of 10 (with illegal acts)	5 of 9 (without illegal acts)	4 of 10 (with illegal acts)	4 of 9 (without illegal acts)
CPGI/PGSI total score (Pearson)	0.654	0.606	0.696	0.671
CPGI/PGSI cut point of 8+ (Kappa)	0.657	0.591	0.659	0.645
SOGS total score (Pearson)	0.677	0.641	0.713	0.666
SOGS cut point of 5+ (Kappa)	0.621	0.569	0.651	0.625
Harmful consequences (Pearson)	0.548	0.516	0.590	0.575
Frequency of gambling (Pearson)	0.299	0.269	0.303	0.296

Next we examined the relationship between each of the DSM criteria and other measures of gambling problems to see if illegal acts played an important role in convergent validity. To do this we conducted a forced entry regression analysis of the DSM criteria with the CPGI/PGSI. The same analysis was also conducted with the SOGS, gambling frequency and harmful consequences. The question of interest was whether illegal acts contributed significantly to the relationship between DSM-IV criteria and these other measures of gambling problems. The results are shown in Table 6. Of particular note: the illegal acts criterion was significantly related to the total score for each of the four measures. Only two other criteria were significant in all four analyses; criterion 2 (gambling with increasing amounts of money) and criterion 6 (chasing).

**Table 6:** Beta coefficients from regression analysis of DSM criteria onto CPGI/PGSI, SOGS, harmful consequences, and gambling frequency

DSM-IV	CPGI/PGSI	SOGS	Harmful consequences	Gambling frequency
Criteria	*n* = 669	*n* = 672	*n* = 661	*n* = 665
Criterion 1	0.108***	0.093**	0.054	0.057
Criterion 2	0.124***	0.122***	0.093**	0.216***
Criterion 3	0.082***	0.115***	0.035	−0.009
Criterion 4	0.001	−0.019	0.095*	0.010
Criterion 5	0.129***	0.146***	0.132***	0.012
Criterion 6	0.205***	0.100***	0.089*	0.176***
Criterion 7	0.268***	0.313***	0.222***	0.036
Criterion 8	0.101***	0.108***	0.080*	0.161***
Criterion 9	0.097**	0.043	0.136***	0.059
Criterion 10	0.042	0.149***	0.031	−0.038
	R^2^ = 0.651	R^2^ = 0.665	R^2^ = 0.455	R^2^ = 0.241

See Table 1 for list of criterion content

* *p* < 0.05, ** *p* < 0.01, *** *p* < 0.001

To examine the importance of illegal acts in terms of severity of disordered gambling, we grouped people into mild (4–5), moderate (6–7) or severe (8–10) gambling disorders with and without the illegal acts criterion. As shown in Table 7, the illegal acts criterion affects the grouping of several individuals. The data indicate that by including illegal acts, more individuals would be identified as having moderate and severe gambling disorders. Interestingly, the illegal acts criteria even increased the number of sub-clinical problem gamblers that were identified.

**Table 7:** Frequency distribution of DSM-5 scores assuming a cut off of four with and without the illegal acts criterion (*N* = 6 76)

	DSM-5 without illegal acts		DSM-5 with illegal acts		DSM-5 with illegal acts combined with lying to others	
	Frequency	%	Frequency	%	Frequency	%
No problem (0)	467	69.1	465	68.8	465	68.8
Sub-clinical (1–3)	146	21.6	142	21.0	144	21.3
Mild (4–5)	36	5.3	37	5.5	40	5.9
Moderate (6–7)	16	2.4	19	2.8	16	2.4
Severe (8–9)	11	1.6	13	1.9	11	1.6

For the DSM-5 with eight there are ten criteria so this has to be taken into account when comparing the models

As noted above, the illegal acts criterion is more often endorsed by disordered gamblers in the correctional system. One option for reducing the possibility of missing some disordered gamblers is if illegal acts are assessed under the “lying to other” criterion (criterion 7). This is a solution explicitly endorsed by DSM-5 (American Psychiatric Association 2013; Grant 2013). In particular they state “these instances of deceit may also include, but are not limited to, covering up illegal behaviors such as forgery, fraud, theft, or embezzlement to obtain money with which to gamble” (American Psychiatric Association 2013, p. 586). To investigate this as an option we conducted an analysis combining illegal acts (criterion 8) and lying to others (criterion 7) to determine if this combination would improve the scale. This entailed scoring the person a 1 if they had endorsed either criterion 7 or criterion 8. It should be noted that this is not identical to what is in DSM-5, where illegal acts are only assessed in terms of the deceit used and not the illegal act itself. This analysis suggested an improvement compared to the DSM-5 without illegal acts. As noted above in Table 2, 63 offenders (9.3 % of the total) were identified as GD without illegal acts and 69 (10.2 %) were identified as GD with illegal acts. By combining criteria 7 and 8, the number of people identified as GD was 67 (9.9 %).

## Discussion

The results of these analyses support the change in the cut score of the DSM from five to four. In these analyses, more offenders were screened in by the reduction of the criteria from five or more to four or more. Petry et al. (2013) found that the change from 5 to 4 criteria resulted in a very small change in prevalence estimates among non-correctional samples. However, the current study indicates that the percentage of offenders identified with a gambling disorder would increase substantially from 7.4 to 9.3 % using a 4 of 9 criteria, representing an increase of 26 %. In addition, the somewhat higher correlations of a cut score of four criteria compared to a cut off of five criteria with external variables such as the SOGS and the CPGI/PGSI suggest that the change in threshold increased the accuracy of the diagnosis within offender populations and reduced the false negative rate. Stinchfield et al. (2013; see also Stinchfield 2003) have argued that a false positive is a more costly diagnostic error from a clinical standpoint.

Previous studies with non-offender populations had found that the elimination of the illegal acts criterion did not substantively affect the prevalence rate of disordered gambling (Petry et al. 2013). In the current study we found that when the illegal acts criterion was removed, the percentage of offenders identified as having a gambling disorder would decrease from 10.2 to 9.3 % (a decrease of 8.7 %). In addition, including the illegal acts criterion yielded higher correlations with measures of convergent validity, including the SOGS and CPGI/PGSI, indicating that the illegal acts criterion increased the accuracy of diagnosis. The regression analyses indicated that the illegal acts criterion contributes substantively to the convergent validity of the DSM criteria with the SOGS and CPGI/PGSI. In fact, in the regression analyses only three criteria (e.g., criterion 2: increasing amounts of money, criterion 6: chasing, and criterion 8: committing illegal acts) were significantly related to all four external criteria (e.g., SOGS, CPGI/PGSI, harmful consequences, and gambling frequency). This finding reinforces the importance of illegal acts criterion for correctional populations and in particular illustrates the importance of illegal acts in determining severity of the disorder. The DSM-5 actually includes a range in severity from mild to medium (Grant 2013). As noted by others, the illegal acts criterion is often endorsed by the most severe cases (Strong and Kahler 2007). The regression analysis suggests that illegal acts may be particularly useful in determining the severity of the client’s problem. Thus the results do not support the elimination of the illegal acts criterion with correctional samples.

The solution of assessing illegal acts under the “lying to others” criterion as stated in DSM-5 (American Psychiatric Association 2013; Grant 2013) to some extent addresses this problem. This option makes sense given that most illegal acts, including fraud which is commonly reported amongst gambling disordered offenders, inherently involve deception. Our results found that the number of missed cases dropped from 6 to only 2 if “illegal acts” was combined with “lying to others.” Thus if clinicians were to inquire about illegal acts when asking clients about examples of lying to others, it appears few individuals with GD would be misclassified.

As with all research there are some limitations. The study did not use a DSM-5 questionnaire, but estimated DSM-5 scores based answers to the DSM-IV. The DSM-IV questionnaire used was one we have used in numerous previous studies (Turner et al. 2006, 2008). Another limitation is that by necessity there is some content overlap between the DSM-IV, CPGI/PGSI, the SOGS and the Harmful Consequences Questionnaire that likely spuriously inflated the correlation and regression coefficients. However, the key item in question in these analyses, “illegal acts”, is unique to the DSM-IV. The SOGS has one item that indirectly addresses an illegal behavior (e.g., “passing bad checks”), but no direct statement about illegal acts. The harmful consequences scale and CPGI/PGSI have no items that specifically ask about illegal acts so the higher correlation coefficient, when “illegal acts” is included in the scale, is not an artefact of overlapping content. However, it is important to note that the results from these correctional samples likely cannot be generalized to general population samples or even clinical samples.

In conclusion, analyses of the changes to GD for DSM-5 found the reduction of the cut score from five to four to be an improvement compared to the DSM-IV. However, for this offender population, the results do not support the elimination of the illegal acts criterion and suggest that it may cause a number of missed diagnoses compared to including the illegal acts criterion. Considering the contrast between these findings and other published studies (Petry et al. 2013; Strong and Kahler 2007; Zimmerman et al. 2006), it would appear that the illegal acts criterion is more relevant for correctional populations than for other populations. In addition, the illegal acts criterion improves the convergent validity of the DSM-5 with other measures of problem gambling, and it would help in the classification of clients into mild, moderate or severe cases. However, subsuming illegal acts under the “lying to others” criterion as stated in DSM-5 does appear to address these problems to some extent.
